# Immunogenicity and Protective Effects of an Ag85B Tuberculosis Subunit Vaccine Formulated with Synthetic TLR4 Agonists in BCG-Boosted Mice

**DOI:** 10.3390/vaccines14030214

**Published:** 2026-02-26

**Authors:** Soo-Min Kim, Jin-Seung Yun, EunJung Shin, Jinhee Lee, You-Jin Kim, Hye-Sook Jeong, Yong Woo Jung, Dokeun Kim

**Affiliations:** 1 Division of Infectious Disease Vaccine Research, Center for Vaccine Research, National Institute of Health, Korea Disease Control and Prevention Agency, 212 Osongsaengmyeong 2-ro, Osong-eup, Heungdeok-gu, Cheongju 28160, Republic of Korea; tnals0903@korea.kr (S.-M.K.); yun0516@korea.kr (J.-S.Y.); yjiney2@korea.kr (Y.-J.K.); jeongnih@korea.kr (H.-S.J.); 2 College of Pharmacy, Korea University, Sejong 30019, Republic of Korea; 3 Bio Research Center, Quratis Inc., Osong 28161, Republic of Korea; ejshin@quratis.com (E.S.); lee8583@quratis.com (J.L.)

**Keywords:** tuberculosis vaccine, subunit vaccine, adjuvant, TLR4 agonist, STING agonist

## Abstract

Background/Objectives: Tuberculosis (TB) remains a major global health challenge, and the Bacillus Calmette–Guérin (BCG) vaccine has limited efficacy against adult pulmonary disease. Protein subunit vaccines are a promising alternative but require strong adjuvants to induce cell-mediated immunity. Synthetic agonists targeting toll-like receptor 4 (TLR4) and stimulators of interferon genes (STINGs) have emerged as effective immunostimulants. Therefore, we aimed to evaluate the immunogenicity and protective efficacy of Ag85B-based subunit vaccines formulated with synthetic TLR4 and STING agonists in a BCG-boosted mouse model. Methods: Three synthetic adjuvants—QTP709-1, QTP709-3, and QTP701—were formulated as oil-in-water emulsions containing distinct surfactant and immunostimulant components. The potential of vaccine formulations to activate dendritic cells (DCs) and elicit Ag85B-specific immune responses, including IgG subclass levels, interferon-γ (IFN-γ) enzyme-linked immunosorbent spots, and polyfunctional T-cell responses, was assessed by flow cytometry. Protective efficacy was evaluated based on pulmonary bacterial burden and histopathology following *Mycobacterium tuberculosis* (M. tb) Erdman challenge. Results: All formulations promoted DC maturation and enhanced antigen-specific immune responses. Each adjuvant elicited strong Ag85B-specific humoral immunity, increased IFN-γ secretion, and polyfunctional CD4^+^ and CD8^+^ T cells co-producing IFN-γ, TNF-α, and interleukin-2. Among them, QTP709-1 was associated with increased levels of chemokine receptor 5-associated chemokines and showed a trend toward reduced lung bacterial burden and histopathological inflammation following M. tb challenge. Conclusions: Synthetic TLR4 and STING agonists were associated with enhanced immunogenicity of TB subunit vaccines and showed evidence of protective potential, with TLR4-based formulations exhibiting more pronounced immunological responses. QTP709-1 exhibited strong immunostimulatory and protective effects, supporting its potential as a candidate adjuvant for next-generation TB vaccines.

## 1. Introduction

Tuberculosis (TB) remains one of the leading infectious diseases worldwide, causing approximately 10 million new cases and 1.5 million deaths annually [1]. Advancements in anti-TB chemotherapy, public health interventions, and improvements in sanitation and living conditions have collectively helped reduce TB incidence and mortality over the past century [2]. However, TB continues to pose a major global health challenge, particularly in high-burden and resource-limited settings [3]. Vaccination remains one of the most effective and sustainable strategies to reduce TB transmission and disease burden. Modeling studies predict that an effective adolescent or adult TB vaccine could significantly reduce global TB incidence and mortality, with the greatest public health impact in high-burden settings [4].

The Bacillus Calmette–Guérin (BCG) vaccine, developed over a century ago, remains the only licensed TB vaccine for human use [5]. Despite its widespread administration, BCG provides limited protection against pulmonary TB and does not reliably prevent *Mycobacterium tuberculosis* (M. tb) infection [6]. Consequently, developing next-generation TB vaccines capable of inducing stronger and more durable immunity has become a global priority [7]. Multiple TB vaccine platforms have been investigated [8], including live attenuated, viral vector-based, protein subunit vaccines, DNA, and mRNA vaccines [9]. For instance, mRNA-based candidates like BNT164 are currently being evaluated in Phase I/II clinical trials to assess their safety and immunogenicity [10]. These platforms allow rapid antigen design and strong in vivo expression, with reported potential to induce Th1-biased cellular immunity, including CD8^+^ T-cell responses important for intracellular M. tb infection [11]. Despite these advantages, mRNA vaccines depend on LNP delivery and stringent cold-chain logistics, which remain key challenges for deployment in high-burden regions [12]. DNA vaccines are stable and cost-efficient, but frequently require assisted delivery methods (e.g., electroporation, gene gun) to achieve sufficient immunogenicity [13]. Among these, protein subunit vaccines are particularly promising because of their established safety, stability, and ability to elicit antigen-specific immune responses [14]. However, subunit vaccines alone often fail to induce sufficient protective immunity, underscoring the need for adjuvants that enhance and sustain the immune response [15].

To overcome these limitations, extensive research has focused on adjuvant-based vaccines [16]. Adjuvants are crucial for improving vaccine efficacy because they promote strong and sustained immune responses [17]. The aluminum salt adjuvant (Alum) is widely used in licensed human vaccines and has an excellent safety profile [18]. However, its limited ability to elicit robust T helper 1 (Th1) and Th17 responses makes it suboptimal for TB vaccines, which primarily rely on cell-mediated immunity [19]. Newer adjuvant systems, such as CAF01, a liposomal adjuvant, have demonstrated the ability to elicit long-lasting M. tb-specific T-cell responses in humans [20]. Similarly, GLA-SE, a synthetic toll-like receptor 4 (TLR4) agonist formulated as a stable emulsion, enhanced both the magnitude and polyfunctionality of antigen-specific T-cell responses in the ID93 subunit vaccine in a first-in-human clinical trial [21]. Moreover, several TB vaccine candidates incorporating immunostimulatory adjuvants are in preclinical and clinical trials and have shown promising immunogenicity and protective efficacy [22,23]. For TB vaccination, adjuvants that enhance Th1 and Th17 immunity are particularly important [24] as these immune pathways support bacterial control and long-term protection [25]. To this end, several studies have explored various adjuvant formulations for TB vaccines, including TLR [26] and stimulator of interferon gene (STING) agonists [27], which activate key innate immune pathways and enhance adaptive immunity. Furthermore, we developed novel variants of these agonists to optimize their immunostimulatory properties and assessed their potential as TB vaccine adjuvants.

Ag85B, a fibronectin-binding protein with mycolyltransferase activity [28], is one of the major antigens secreted by M. tb. It plays a pivotal role in cell wall synthesis and serves as a dominant target of host T-cell responses, eliciting robust Th1-type immune activation [29]. Patients with latent and active tuberculosis have demonstrated Ag85B-specific humoral immune responses [30], indicating that Ag85B remains immunologically accessible throughout the infection process. The immunogenicity of Ag85B has also been demonstrated by the presence of both humoral and cell-mediated antigen-specific immune responses [31]. Because of its strong immunogenicity and conservation across mycobacterial species, it is a key antigen for developing subunit vaccines. Ag85B was selected as the target antigen because it contains well-characterized immunodominant T-cell epitopes that consistently induce robust CD4^+^ Th1-biased responses, which are critical for controlling intracellular M. tb infection [32]. Furthermore, its clinical relevance is underscored by its inclusion in several vaccine candidates currently in clinical trials, such as H56:IC31 [33] and GamTBvac [34].

TLR4 agonists, such as synthetic monophosphoryl lipid A (MPLA), are potent immunostimulatory agents that enhance vaccine-induced immunity by activating innate immune signaling pathways [35]. They play a crucial role in activating antigen-presenting cells (APCs), including macrophages and dendritic cells (DCs) [36], which in turn promote Th1 polarization [37] and interferon-γ (IFN-γ) production [38]. TLR4 agonists promote Th1 immunity, enhance antibody production, and facilitate antigen uptake, contributing to a robust and sustained adaptive immune response [39]. Therefore, several synthetic and naturally derived TLR4 agonists have been developed and incorporated into vaccine formulations to improve immunogenicity and long-term protection.

Similarly, STING agonists activate the cyclic GMP–AMP synthase (cGAS)–STING pathway and are key modulators of innate and adaptive immunity [40]. This pathway detects cytosolic deoxyribonucleic acid (DNA) and triggers a type I interferon response [41], leading to DC maturation and migration [42], cross-priming of cluster of differentiation (CD)8^+^ T cells, and activation of natural killer cells, thereby reinforcing T cell-mediated immunity [41]. STING agonists also promote sustained immune surveillance [43] and elicit Th17 responses [27], both of which are crucial for long-term protection against TB. Given these properties, TLR4 and STING agonists are actively being investigated as immunostimulants in next-generation TB vaccines [44].

In this study, we aimed to investigate the immunogenicity and protective efficacy of Ag85B, a well-characterized TB antigen, formulated with synthetic TLR4 and STING agonists as adjuvants. Specifically, we aimed to evaluate the ability of the vaccine formulations to induce antigen-specific humoral and cellular immune responses, including immunoglobulin (Ig)G production, cytokine secretion, and activation of polyfunctional T cells. Furthermore, we examined their protective efficacy in a murine TB model by quantifying bacterial burden and lung pathology after challenge with the virulent M. tb Erdman strain. The findings contribute to identifying promising adjuvant platforms for the development of next-generation TB vaccines.

## 2. Materials and Methods

### 2.1. Materials

Ag85B protein was acquired from Abcam (Cambridge, UK). RPMI-1640 medium, fetal bovine serum (FBS), penicillin/streptomycin (P/S), and Dulbecco’s phosphate-buffered saline (DPBS) were sourced from Gibco (Waltham, MA, USA). Reagents used for immunological assays were procured from BD Biosciences (San Diego, CA, USA).

### 2.2. Adjuvants and Formulations

MPLA, a synthetic TLR4 agonist, was purchased from Avanti Polar Lipids (Snaith, UK). The TLR4-based adjuvants QTP709-1 and QTP709-3 were formulated as oil-in-water emulsions by mixing an oil and aqueous phase, whereas MPLA was dissolved in squalene (Sigma-Aldrich, St. Louis, MO, USA). The two formulations were distinguished by their phospholipid composition: QTP709-1 was formulated using 1,2-dimyristoyl-sn-glycero-3-phosphocholine (DMPC) (LIPOID GmbH, Ludwigshafen, Germany), whereas QTP709-3 was formulated using a combination of sorbitan monostearate (Span 60; Sigma-Aldrich, St. Louis, MO, USA) and polysorbate 80 (Tween 80) (Spectrum, New Brunswick, NJ, USA). Stable emulsions were produced by mixing the aqueous and oil phases containing key components, including the TLR4 agonist, squalene, and phospholipids, using a high-shear mixer, followed by homogenization with a microfluidizer [45]. The STING agonist QTP701 adjuvant was prepared by mixing cyclic di-GMP (c-di-GMP) (Sigma-Aldrich, MO, USA) with a conventional stable emulsion formulation lacking MPLA. All adjuvants were manufactured and supplied by Quratis (Osong, Korea) and quality-controlled in accordance with Quratis’ internal regulatory standards. The analytical properties of the three QTP oil-in-water (O/W) adjuvant emulsions, including emulsion homogeneity and batch-to-batch reproducibility, are summarized in Supplementary Table S1.

The adjuvants were packaged in 2 mL glass vials at 0.1 mg/mL and stored at 4 °C until use. For immunization, mice were administered 1 µg recombinant Ag85B antigen formulated with 5 µg of each adjuvant (QTP709-1, QTP709-3, and QTP701).

### 2.3. Bacteria

BCG (Pasteur strain 1173P2) was supplied by the Korea Disease Control and Prevention Agency (KDCA), and M. tb Erdman strain was acquired from the American Type Culture Collection (Manassas, VA, USA). Bacteria were maintained on Middlebrook 7H10 agar supplemented with 10% oleic acid–albumin–dextrose–catalase (OADC; BD Biosciences, Dickinson, Sparks, MD, USA) and 0.5% glycerol and incubated 3–4 weeks at 37 °C. Middlebrook 7H9 broth medium (Difco, Detroit, MI, USA) supplemented with 10% albumin–dextrose–catalase (ADC; BD Biosciences), 0.5% glycerol, and 0.05% Tween 80 (Sigma-Aldrich, Germany) and was used for bacterial culture. To obtain single-cell suspensions, mycobacterial cells were collected by centrifugation at 10,000 *g* for 20 min followed by two washes with PBS (pH 7.2). To generate single-cell suspensions, pellets were passed sequentially through 70 μm to 10 μm pore-size filters (Millipore Corp., Burlington, MA, USA), to reduce clumping and remove aggregates. Cell working stocks were prepared and stored at −80 °C until further use. Bacterial loads were quantified as colony-forming units (CFUs) by plating serial dilutions onto 7H10 agar plates to assess bacterial burden.

### 2.4. Analysis of Antigen Presentation In Vitro

#### 2.4.1. Generation of Bone Marrow-Derived DCs (BMDCs)

BMDCs were differentiated from murine bone marrow cells of euthanized mice. Femur bones were aseptically removed and disinfected by immersion in 75% ethanol for 1 min. After rinsing thrice with DPBS, both ends of each femur were removed, and the bone marrow cells were flushed out with DPBS using a syringe and needle. Red blood cells were removed from the isolated BMDCs following exposure to ACK Lysis Buffer (Gibco, Grand Island, NY, USA). After washing with RPMI-1640 medium supplemented with 10% FBS and 1% P/S, bone marrow cells were resuspended at a density of 1 × 10^6^ cells/mL in complete RPMI supplemented with 20 ng/mL granulocyte-macrophage colony-stimulating factor (GM-CSF; Peprotech, London, UK) and 10 ng/mL interleukin-4 (IL-4; PeproTech), and maintained for 10 days at 37 °C in a humidified incubator with CO_2_. Non-adherent cells were removed, and the culture medium containing GM-CSF and IL-4 was refreshed every 2 days.

#### 2.4.2. BMDCs Pulsed with Antigen and Adjuvants

Immature DCs were harvested and seeded at a density of 1 × 10^5^ cells/mL in a 6-well plate. Enriched immature DCs were incubated for 48 h at 37 °C in a CO_2_ incubator in the presence of Ag85B (10 µg/mL) together with 50 µg/mL of each adjuvant (QTP709-1, QTP709-3, and QTP701).

#### 2.4.3. T-Cell Activation Using Pulsed DC

Lymphocytes were isolated from the spleen cells of Ag85B-immunized C57BL/6 mice, labeled with Carboxyfluorescein succinimidyl ester (CFSE), and co-cultured with DCs pre-exposed to the antigen and adjuvants. The DC:lymphocyte ratio was 1:20. After 5 days incubation period, culture supernatants were harvested and cryopreserved at −80 °C for subsequent analyses, while cells were collected, subjected to surface marker and intracellular cytokine staining, and evaluated by flow cytometry.

#### 2.4.4. Flow Cytometry Analysis

After antigen pulsing, cells were processed for flow cytometric analysis of surface marker expression. For surface staining, 1 × 10^6^ cells were washed and incubated for 30 min on ice with Fc receptor-blocking reagent (FcγIII/IIR Ab; BD PharMingen, San Diego, CA, USA). Blocked cells were subsequently stained with a panel of fluorochrome-conjugated monoclonal antibodies (mAbs; BD Pharmingen), including PE-labeled anti-CD11c, FITC-labeled anti-CD80, BV421-labeled anti-CD86, APC-labeled anti-MHC class II, FITC-labeled anti-CD4, and FITC-labeled anti-CD8. After washing, cells were resuspended in sorting buffer and analyzed using a CytoFLEX flow cytometer (Beckman Coulter, Indianapolis, IN, USA). At least 10,000 gated events were recorded per sample, and data were processed using FlowJo software (version 10.9.0; Tree Star, Ashland, OR, USA).

#### 2.4.5. Cytokine Complex Multiplex

Cytokine profiles in supernatants from pulsed BMDC and T-cell activation assays were analyzed using a Bio-Plex MAGPIX Multiplex Reader system (Bio-Rad, Milan, Italy) equipped with Bio-Plex Manager software v6.1 (BioRad) according to the manufacturer’s instructions. Measurements were performed in triplicate using 50 µL samples and a Bio-Plex Pro Mouse Cytokine 23-plex assay kit (BioRad). Standard curves were optimized, and analyte concentrations were calculated using Bio-Plex Manager software v6.1 (BioRad).

### 2.5. Animal Experiments

#### 2.5.1. Ethics Statement

All animal experiments were conducted in compliance with the guidelines of the Institutional Animal Care and Use Committee and were approved by the Korea Disease Control and Prevention Agency (approval number: KDCA-IACUC-23-016).

#### 2.5.2. Animal Husbandry

Four-week-old female specific pathogen-free (SPF) C57BL/6 mice were purchased from ORIENTBIO Inc. (Seongnam, Republic of Korea). All mice were maintained under SPF conditions in temperature-, relative humidity-, and light-controlled facilities within the Animal Biosafety Level (ABSL)-II and ABL-III laboratories of the KDCA. The animals had free access to dry pellets and sterile water. The mice were anesthetized and euthanized using CO_2_ inhalation at the end of the study.

#### 2.5.3. Vaccination Protocol

All mice were immunized after 1 week of adaptation. BCG was subcutaneously vaccinated once with 2 × 10^5^ CFUs resuspended in 0.1 mL PBS. Six weeks later, mice were immunized with subunit vaccine candidates formulated with adjuvants. These subunit vaccine candidates were administered subcutaneously in duplicate at 3-week intervals. Spleen and lung cells were harvested two weeks after the final immunization and processed for immunogenicity analysis.

#### 2.5.4. Experimental Challenge

Six weeks after the final immunization, 5 × 10^6^ CFU of the M. tb strain Erdman in 3 mL was performed with a Glass-Col airborne infection system (Terre Haute, IN, USA). Each mouse was exposed to M. tb strain within an inhalation chamber for a 60 min period, under conditions calibrated to deliver a predetermined infectious dose. To confirm the initial bacterial burden, lung deposition corresponding to approximately 50–100 CFUs was achieved. Bacterial loads were assessed by enumerating colonies after plating organ homogenates on 7H10 agar OADC plates.

### 2.6. Anti-Ag85b-Specific IgG Enzyme-Linked Immunosorbent Assay (ELISA)

Serum levels of Ag85B-specific IgG (H + L), IgG1, and IgG2c were evaluated as described previously. Briefly, 96-well plates (Thermo Fisher Scientific, Waltham, MA, USA) were coated with 1 μg/mL Ag85B for 18 h at 4 °C. Serum samples were diluted 1:100 in PBS supplemented with 3% BSA and incubated for 2 h at 37 °C. After washing the samples, HRP-conjugated secondary antibodies specific for IgG (H + L), IgG1, or IgG2c (Thermo Fisher Scientific) were applied. Following chromogenic development, optical density was recorded at 450 nm on a SpectraMax i3x spectrophotometer (Molecular Devices, San Jose, CA, USA) within 20 min after reaction termination.

### 2.7. Assessment of Cellular Immune Responses

#### 2.7.1. Lymphocyte Preparation

To evaluate vaccine-induced immunogenicity, splenocytes and lung lymphocytes were isolated from each group of mice. For single-cell preparation, spleens and lungs were aseptically harvested and pooled within each group. Spleens were mechanically dissociated with a gentleMACS Tissue Dissociator (Miltenyi Biotec, Bergisch Gladbach, Germany), washed with RPMI-1640 medium supplemented with 10% FBS and 1% penicillin–streptomycin (P/S), and passed through a 70 μm nylon mesh cell strainer (BD Biosciences). The filtered splenocytes were treated with ACK Lysis Buffer for 5 min at 24 ± 2 °C to remove erythrocytes. The resulting single-cell suspension was rinsed twice with RPMI-1640 medium and prepared for downstream analyses.

For single-cell preparation, lungs were aseptically harvested and subjected to enzymatic digestion at 37 °C for 1 h in RPMI-1640 digestion medium supplemented with collagenase D and DNase I (Roche, Basel, Switzerland). Enzyme-treated lung tissues were processed by density centrifugation using a Lymphoprep gradient (STEMCELL Technologies, Vancouver, BC, Canada). The resulting lung cell suspension was filtered through a 70 μm cell strainer and adjusted in complete RPMI to generate a single-cell preparation of lung-derived lymphocytes.

#### 2.7.2. Mouse IFN-γ ELISpot Assays

IFN-γ–secreting cells were assessed using an enzyme-linked immunosorbent spot (ELISpot) assay in splenocytes and lung-derived lymphocytes. The assay was conducted using a commercial mouse IFN-γ ELISpot kit (R&D Systems, Minneapolis, MN, USA) according to the manufacturer’s instructions. Cells were seeded at 5 × 10^6^ cells per well and stimulated with Ag85B protein (1 μg/mL) for 36 h at 37 °C in anti–IFN-γ antibody–coated plates. The plates were washed with wash buffer, then incubated with a biotinylated anti-IFN-γ antibody at room temperature (24 ± 2 °C) for 2 h. Thereafter, alkaline phosphatase-conjugated streptavidin was added to each well and incubated at room temperature for 2 h. Finally, the reaction was developed using a 3-amino-9-ethylcarbazole (ACE) solution for 60 min at RT, and the resulting spots were quantified using a CTL Immunospot reader (Cleveland, OH, USA). The results are expressed as mean values of triplicate wells for each group.

#### 2.7.3. Quantification of Cytokines

A single-cell suspension at a density of 5 × 10^6^ cells per well was stimulated with Ag85B for 36 h at 37 °C, after which the supernatants were collected to measure cytokine expression. The assays were performed in triplicate for each group using the Bio-Plex Pro mouse cytokine and chemokine panel (Bio-Rad Laboratories), customized to quantify IL-2, IL-12p40, IL-17, monocyte chemoattractant protein-1 (MCP-1), macrophage inflammatory protein-1 alpha (MIP-1α) and beta (MIP-1β), keratinocyte-derived cytokine (KC), GM-CSF, and IL-1β, according to the manufacturer’s instructions. The data were acquired using the Bio-Plex MAGPIX reader (version 5.0) software (Bio-Rad Laboratories) and analyzed as mean values.

#### 2.7.4. Flow Cytometry and Intracellular Cytokine Staining

Lung-derived lymphocytes were plated at 5 × 10^6^ cells/mL and exposed to Ag85B (1 μg/mL) for 10 h at 37 °C with GolgiStop (BD Biosciences). After incubation, cells were rinsed in Stain Buffer (BD Biosciences) and subjected to surface staining with FITC-conjugated anti-CD3, V450-conjugated anti-CD4, and PerCP-Cy5.5-conjugated anti-CD8a antibodies for 30 min at 4 °C. The cells were subsequently fixed and permeabilized with a Cytofix/Cytoperm kit (BD Biosciences). Intracellular cytokines were identified following staining with APC-conjugated anti-TNF-α, PE-conjugated anti-IL-2, and PE-Cy7-conjugated anti-IFN-γ antibodies in permeabilization buffer. All antibodies were purchased from BD Biosciences, unless otherwise stated. The stained cells were analyzed using a CytoFLEX flow cytometer (Beckman Coulter, Brea, CA, USA), and the data were processed with FlowJo software (version 10.9.0; Tree Star, Ashland, OR, USA).

### 2.8. Bacterial Burden Assay and Histopathology

Protective efficacy was assessed by collecting lungs from infected mice at 6 weeks post-infection. All lung lobes except the right superior lobe were mechanically processed in 3 mL PBS, and resulting homogenates were serially diluted and applied to Middlebrook 7H10 agar plates (Becton Dickinson, Franklin Lakes, NJ, USA) with the addition of 10% OADC. Bacterial colonies were enumerated following incubation at 37 °C for 3–4 weeks. For histopathological analysis, the right superior lung lobe was collected, fixed overnight in 10% neutral-buffered formalin (Sigma-Aldrich, USA), then processed for paraffin embedding, sectioning, and hematoxylin and eosin (H&E) staining. A certified pathologist performed a detailed evaluation of the lung injury and inflammatory lesions. The assessment included identifying and delineating granulomatous regions characterized by cellular infiltration, necrosis, and disruption of the lung parenchymal structure. Granulomatous inflammatory areas were manually delineated by a pathologist on whole-slide images, and the extent of inflammation was quantified with ImageJ software (version 1.54k; National Institutes of Health, Bethesda, MD, USA). The affected area was reported as the proportion of granulomatous lesions within the overall lung tissue area, defined as the percentage of granulomatous inflammation.

### 2.9. Statistical Analyses

All experiments were repeated at least thrice with consistent results. In all analyses, the levels of significance for comparisons between samples were determined using Dunnett’s multiple-comparison test or an unpaired t-test. Statistical analyses were conducted with GraphPad Prism version 9.00 for Windows (GraphPad Software, La Jolla, CA, USA). Comparisons between two groups were conducted using an unpaired *t*-test, while analyses involving three or more groups were performed by one-way analysis of variance followed by Dunnett’s multiple-comparison test. Graphs display data as mean ± standard error of the mean (SEM). Statistical significance was defined as a *p*-value < 0.05. All experiments were performed independently at least three times, and results reflect pooled data from these replicates.

## 3. Results

### 3.1. Formulation of Synthetic TLR4 and STING-Based Oil-in-Water Emulsions

**Figure 1:**
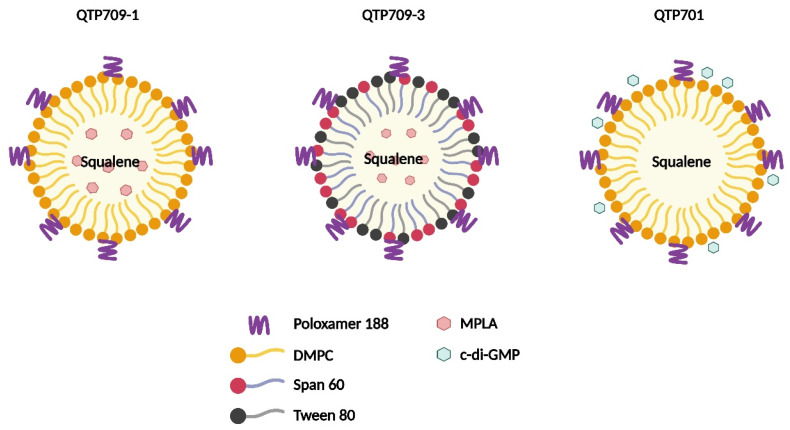
Formulation profiles of synthetic emulsified adjuvants. Three oil-in-water emulsion-based adjuvants—QTP709-1, QTP709-3, and QTP701—were formulated with distinct component profiles. QTP709-1 and QTP709-3 both contained monophosphoryl lipid A (MPLA) and squalene, but differed in their surfactant systems: QTP709-1 included 1,2-dimyristoyl-sn-glycero-3-phosphocholine (DMPC), while QTP709-3 contained Span 60 and Tween 80. The QTP701 formulation excluded MPLA and incorporated the STING agonist cyclic di-GMP in a conventional oil-in-water emulsion base. These adjuvants were used to evaluate vaccine-induced immune responses.

In this study, three adjuvants—QTP709-1, QTP709-3, and QTP701—were formulated as oil-in-water emulsions (Figure 1). Both QTP709-1 and QTP709-3 were formulated as oil-in-water emulsions containing MPLA and squalene, whereas their surfactant compositions differed. The QTP701-based emulsion lacked MPLA and instead incorporated cyclic di-GMP into a conventional oil-in-water base. Subsequently, these formulations were used in vaccine studies to assess their immunological effects.

### 3.2. Synthetic TLR4 and STING Agonists Induce Phenotypic Maturation of Innate Immune Cells and Promote T-Cell Activation Through Adaptive Immune Responses

**Figure 2:**
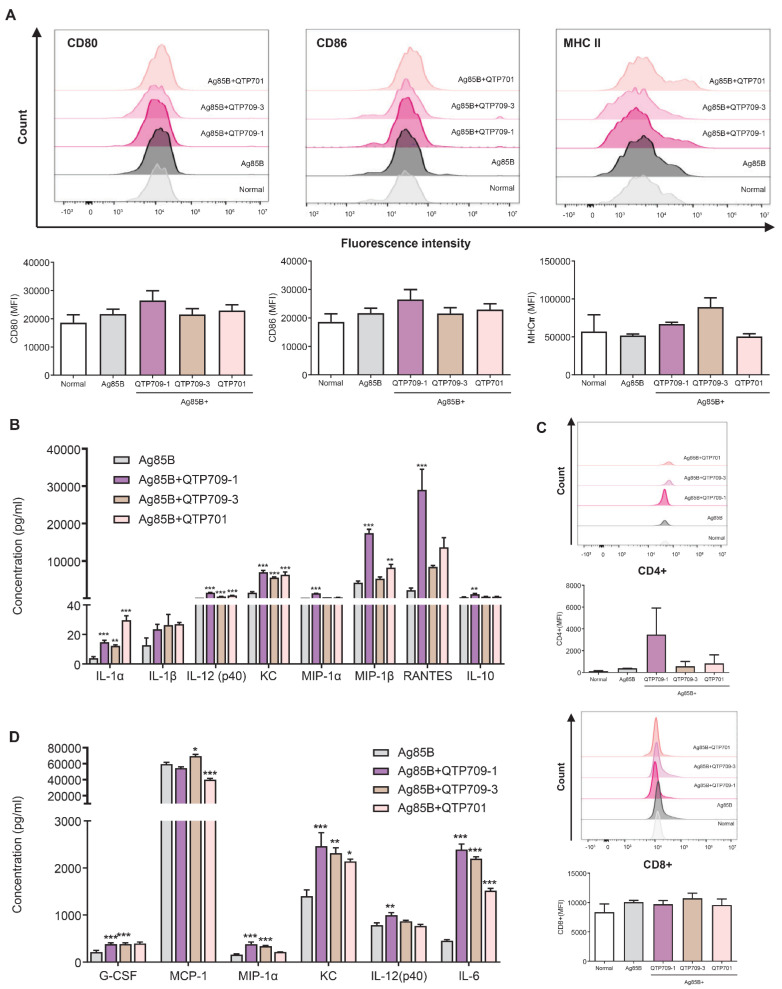
Vaccine adjuvants promoted the phenotypic maturation of dendritic cells (DCs) and enhanced adaptive immune responses by activating T cells and inducing cytokines. (**A**) Bone marrow-derived DCs were stimulated for 24 h with Ag85B protein formulated with various adjuvants. Cells were gated on CD11c^+^ populations, and surface expression of CD80, CD86, and MHC-II was assessed using flow cytometry. Mean fluorescence intensity (MFI) values for individual markers are summarized in bar graphs. (**B**) Levels of IL-1α, IL-1β, IL-12(p40), KC, MCP-1, MIP-1α, RANTES, and IL-10 in culture supernatants measured using the Luminex MAGPIX Multiplex Assay with a Bio-Rad 23-Plex Kit. (**C**) The relative abundance of CD4^+^ and CD8^+^ T-cell populations was determined from cytometry-derived data. DCs following antigen–adjuvant exposure were combined with naïve splenic T cells at a 1:20 DC-to–T cell ratio and maintained for 72 h. (**D**) Cytokine concentrations (G-CSF, MCP-1, MIP-1α, KC, IL-12(p40), and IL-6) in co-culture supernatants were quantified using the Luminex MAGPIX Multiplex Assay. Bar graphs show mean ± SEM. Statistical significance was determined by comparison with the Ag85B group. * *p* < 0.05, ** *p* < 0.01, *** *p* < 0.001. Representative data from one of three independent experiments are shown.

Adjuvants contribute to improved vaccine efficacy by activating innate immune responses [46]. Accordingly, we examined the in vitro activation of BMDCs and evaluated their capacity to induce T-cell responses following exposure to TB vaccine candidates formulated with synthetic adjuvants. The expression of the co-stimulatory molecules, including CD80, CD86, and MHC class II, in DCs was assessed using flow cytometry. All adjuvant-treated groups exhibited increased fluorescence intensity compared with the control group. Notably, QTP709-1 showed a trend toward increased expression of CD80 and CD86, suggesting enhanced DC phenotypic maturation; however, these differences were not statistically significant relative to normal (Figure 2A).

Functional maturation was further supported by cytokine profiling of DC culture supernatants (Figure 2B). Pro-inflammatory cytokines associated with early innate signaling, such as IL-1α, IL-1β, IL-12(p70), and KC were significantly increased following adjuvant treatment. Among these, IL-12(p70), a key driver of Th1 polarization, was most strongly induced by the QTP709-1-based formulations. Furthermore, the chemokines involved in T-cell and monocyte recruitment via the CCR5 signaling axis, including MIP-1α, MIP-1β, and RANTES, were significantly elevated in the QTP709-1 group, suggesting enhanced immune cell trafficking and priming. Anti-inflammatory cytokines, such as IL-10, showed a moderate increase only in certain groups, suggesting a balanced immunomodulatory effect.

In co-culture assays with splenic T-cells, DCs pulsed with QTP709-1-adjuvanted vaccines induced the most robust CD4^+^ T cell activation, whereas CD8^+^ T-cell activation remained comparable across all adjuvanted groups (Figure 2C). Consistent with these functional outcomes, morphological analyses of the co-culture assays revealed structural changes after adjuvant stimulation (Figure S1). DCs treated with Ag85B alone or without adjuvants maintained a round and immature morphology. In contrast, DCs stimulated with adjuvant formulations, particularly QTP709-1, exhibited extended dendritic processes and increased cell spreading, which are hallmarks of a mature, activated DC phenotype [47]. These morphological features aligned with the enhanced antigen-presenting function and T-cell stimulatory capacity observed in the QTP709-1 group.

Furthermore, cytokine analysis of co-culture supernatants (Figure 2D) revealed elevated levels of innate immunity-associated cytokines, including G-CSF, MCP-1, and KC, in adjuvant-containing formulations relative to the Ag85B-only condition. Among these, MCP-1 is a key chemokine that mediates the recruitment and infiltration of monocytes and macrophages to sites of immune activation, suggesting enhanced mobilization of APCs in response to adjuvant stimulation. Moreover, IL-6 and IL-12(p40) levels were significantly elevated across vaccine formulations incorporating adjuvants, relative to both normal controls and the Ag85B-only condition. IL-6 is a central mediator that bridges innate and adaptive immunity by promoting T-cell differentiation [48]. The elevated IL-12(p40) levels were consistent with the induction of Th1-type immune responses [49]. These findings suggest that adjuvant-mediated DC activation facilitates both innate immune cell recruitment and adaptive T-cell priming.

### 3.3. Improvement of Ag85B-Specific Immune Responses in BCG-Primed Adjuvanted Vaccine Candidates

**Figure 3:**
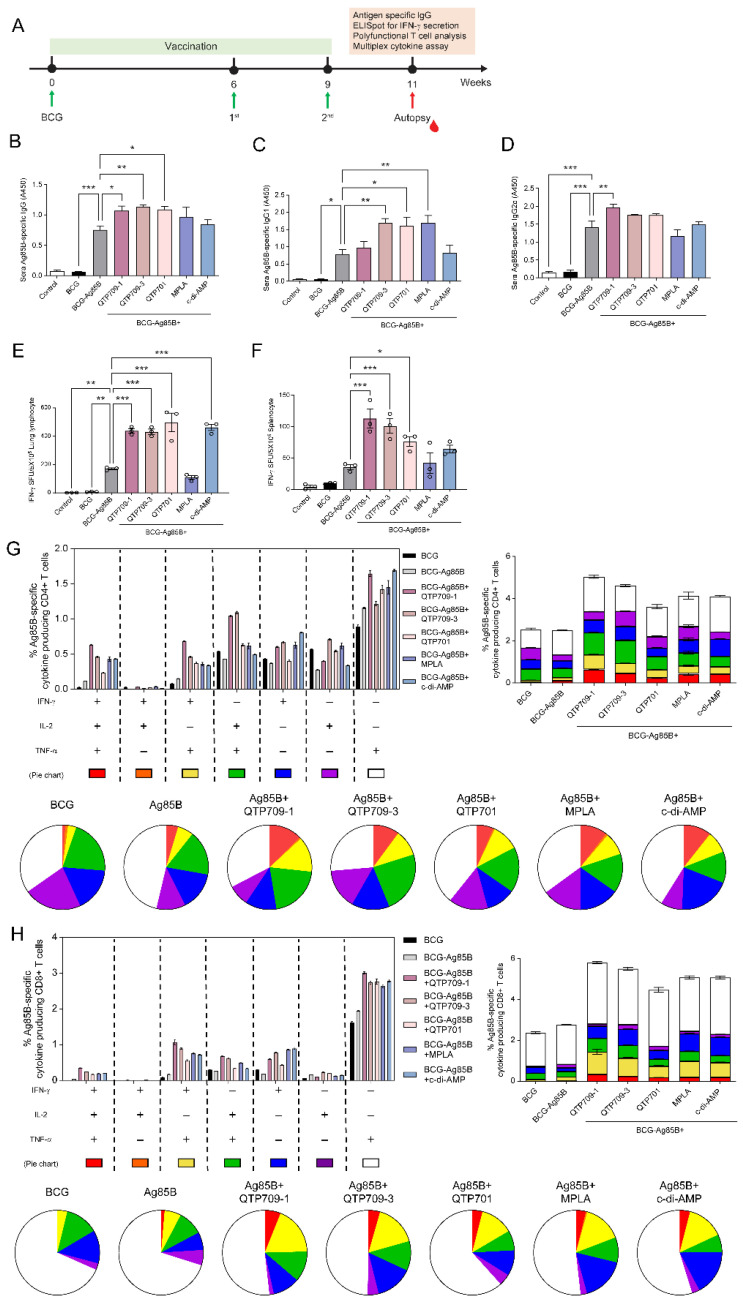
Evaluation of immune responses induced by diverse adjuvanted subunit vaccine in BCG-primed mice. The immunogenicity of Ag85B protein-adjuvanted vaccine candidates was evaluated in C57BL/6 mice (*n* = 5). (**A**) Overview of the experimental workflow. Ag85B-specific antibody responses were assessed by ELISA two weeks following the last immunization, including (**B**) total IgG (H + L), (**C**) IgG1, and (**D**) IgG2c across mice receiving different vaccine formulations. IFN-γ–secreting cell frequencies were determined by ELISpot-based analysis in (**E**) lung lymphocytes and (**F**) splenocytes following re-stimulation with Ag85B (1 μg/mL). Lung lymphocytes collected from vaccinated mice were incubated with Ag85B at 37 °C for 10 h in the presence of Golgi-Stop. Upon stimulation, polyfunctional T-cell responses were analyzed by flow cytometry, assessing the frequency of CD4^+^ T cells producing IFN-γ, TNF-α, and IL-2 (**G**) and the counts of Ag85B-specific, multifunctional CD8^+^ T cells expressing IFN-γ, TNF-α, and IL-2 (**H**). Data are presented as mean ± SEM derived from three measurements per group. Statistical significance was evaluated relative to Ag85B-immunized mice, with * *p* < 0.05, ** *p* < 0.01, and *** *p* < 0.001.

Humoral and cellular immune responses elicited by adjuvanted vaccine candidates were examined two weeks after the last immunization (Figure 3A). Ag85B-specific immune responses were quantified by measuring serum IgG levels, IFN-γ secretion, and polyfunctional T-cell responses. Ag85B-specific antibody titers were measured using ELISA, analyzing total IgG (H + L), IgG1, and IgG2c subclasses to assess humoral immunity. All adjuvanted-immunized groups showed significantly higher IgG (H + L) and IgG1 levels than the Ag85B-only groups. Among the vaccine formulations, the QTP709-3-adjuvanted group elicited the highest IgG (H + L) and IgG1 responses, followed by the QTP701-adjuvanted group (Figure 3B,C). In a similar manner, IgG2c levels were significantly elevated across vaccine formulations containing adjuvants, relative to Ag85B alone. Among these, the QTP709-1-adjuvanted group elicited the highest IgG2c response (Figure 3D), indicating a strong antigen-specific humoral immune response with a Th1 bias [50]. The observed differences in IgG and IgG subclass titers among the vaccine candidates were significant, highlighting the differential immunogenicity of each formulation.

Antigen-specific cellular immunity was evaluated by IFN-γ ELISpot analysis using lung-derived lymphocytes and splenocytes after Ag85B re-stimulation. All vaccine candidates significantly increased IFN-γ secretion compared with Ag85B. Among them, QTP701 induced the highest IFN-γ production in lung lymphocytes (Figure 3E), followed by QTP709-1 and QTP709-3. In splenocytes, QTP709-1 elicited the highest IFN-γ production, followed by the other adjuvants compared with the Ag85B group (Figure 3F). Significant differences in IFN-γ levels were observed across the vaccine groups.

Th1-related immune activity in the lungs was further explored through analysis of polyfunctional T cells. Polyfunctional CD4^+^ and CD8^+^ T-cell populations were characterized by the concurrent production of IFN-γ, TNF-α, and IL-2 as determined from cytometric data. Vaccinated mice showed increased frequencies of polyfunctional T cells relative to Ag85B-immunized mice, with the QTP709-1 group displaying the greatest proportion of triple-positive (IFN-γ^+^/TNF-α^+^/IL-2^+^) CD4^+^ and CD8^+^ T cells, followed in magnitude by the QTP709-3 group (Figure 3G,H).

Ag85B-specific humoral and cellular immune responses were enhanced across all three adjuvant-containing vaccine formulations, QTP709-1, QTP709-3, and QTP701. QTP709-1 demonstrated the highest immune-inducing capacity, as evidenced by elevated antigen-specific IgG levels, increased IFN-γ secretion, and a greater frequency of polyfunctional T cells.

### 3.4. Integrated Innate and Adaptive Immune Responses Elicited by Synthetic Adjuvants

**Figure 4:**
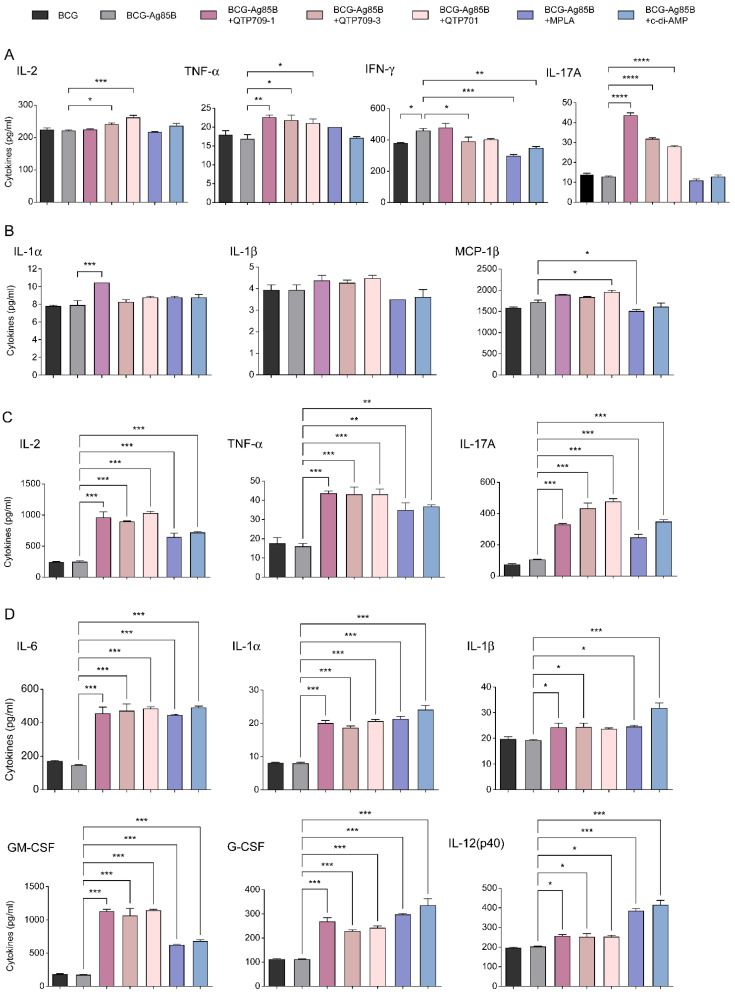
Cytokine secretion in splenocytes and lung lymphocytes following adjuvanted subunit vaccine immunization in BCG-primed mice. Cytokine levels were measured using the Luminex MAGPIX Multiplex Assay system with a Bio-Rad 23-Plex Assay Kit. Cytokine secretion was assessed in the supernatants of lung lymphocytes (**A**,**B**) and splenocytes (**C**,**D**) stimulated with Ag85B protein for 36 h. Cytokine secretion from lung lymphocytes was measured for Th1 cytokines (IL-2, TNF-α, and IFN-γ), Th17 cytokine (IL-17A), and adaptive immunity cytokines (IL-1α, MCP-1β, and IL-1β). Similarly, cytokine secretion from splenocytes was analyzed, including Th1 cytokines (IL-2 and TNF-α), Th17 cytokine (IL-17A), and adaptive immunity cytokines (IL-6, IL-1α, IL-1β, IL-12p40, GM-CSF, and G-CSF). Data are presented as mean ± SEM. Statistical significance was determined by comparing to the Ag85B group: * *p* < 0.05, ** *p* < 0.01, *** *p* < 0.001, **** *p* < 0.0001.

Cytokine profiles in lung- and spleen-derived lymphocytes were examined to characterize immune responses triggered by synthetic adjuvant formulations following vaccination. Cytokine levels in the supernatant were quantified to evaluate Ag85B-specific immune responses. Lung lymphocytes from the vaccinated group exhibited distinct Th1- and Th17-associated cytokine secretion compared with those from the BCG group (Figure 4A). TNF-α, which plays an important role in Th1-associated macrophage activation and adaptive immune responses [51], exhibited significantly increased levels across all vaccine groups, with the QTP709-1-adjuvanted group displaying the greatest TNF-α production. Similarly, IL-17A, a signature Th17 cytokine essential for neutrophil recruitment and mucosal immunity [52], was significantly elevated in all vaccine groups, again with the QTP709-1-adjuvanted group producing the highest levels. These increases suggest that TLR4-agonist-adjuvanted vaccines effectively stimulate Th1 and Th17 responses in the pulmonary environment. Furthermore, IL-17A secretion was observed in all vaccinated groups, with the highest levels observed in the QTP709-1-adjuvanted group, reinforcing its role in Th17-mediated immunity and neutrophil recruitment. Furthermore, QTP709-1-immunized mice exhibited increased levels of adaptive immunity cytokines (IL-1α, IL-1β, and MCP-1 β) compared with the BCG and Ag85B groups (Figure 4B).

Compared with lung lymphocytes, splenocytes exhibited stronger Th1 and Th17 cells (Figure 4C), suggesting more significant systemic immune activation. Th1 cytokines, including TNF-α and IL-2, were significantly elevated in all vaccinated mice, exceeding levels observed in the BCG and Ag85B groups. Furthermore, IL-17A, a hallmark Th17 cytokine, was significantly upregulated. Previous studies have shown that IL-17A directly regulates both innate and adaptive immunity during mycobacterial infection. Cytokine secretion in splenocytes was generally higher than that in lung lymphocytes, indicating a robust systemic immune response in the spleen. TLR4- and STING-agonist adjuvanted vaccines significantly increased Th1 and Th17 cytokine levels, highlighting their strong immunogenic potential.

Furthermore, all vaccinated mice exhibited significantly increased levels of adaptive immunity cytokines (IL-6, IL-1α, IL-1β, IL-12(p40), GM-CSF, and G-CSF) compared with the BCG and Ag85B-immunized mice (Figure 4D). These cytokines collectively enhance the function of the spleen as a central immune organ, facilitating both innate and adaptive immune responses during infection. These findings suggest that adjuvanted vaccine formulations effectively enhance both pro-inflammatory and Th1/Th17-mediated immune responses, which may contribute to improved protection against tuberculosis.

### 3.5. Attenuation of Pulmonary Bacterial Load and Inflammatory Pathology by Adjuvanted Vaccine Formulations in Mice Challenged with Mycobacterium tuberculosis Erdman Strain

**Figure 5:**
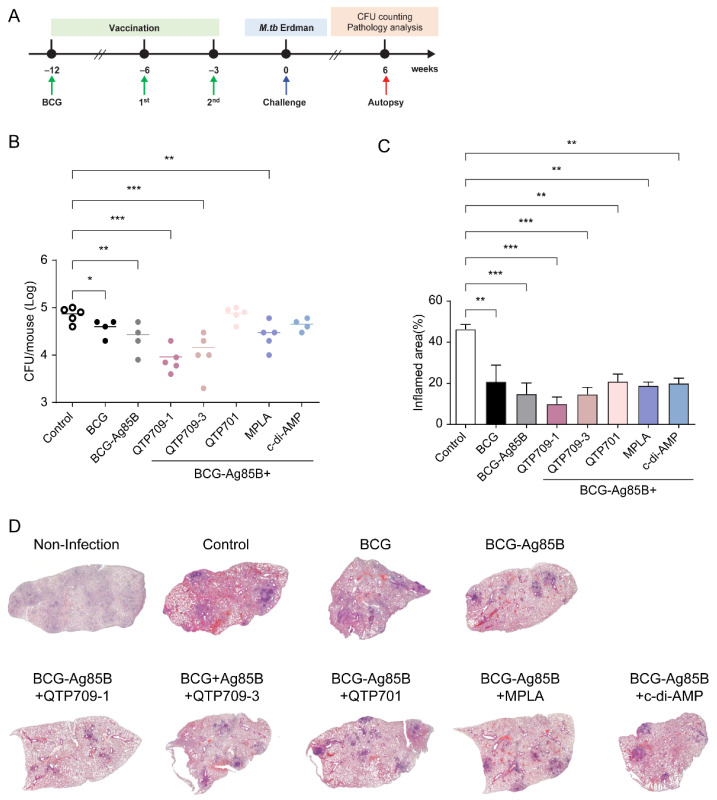
Reduction in *M. tuberculosis* burden and lung inflammation by TB vaccine candidates. The protective efficacy of Ag85B protein-adjuvanted vaccine candidates was evaluated in C57BL/6 mice (*n* = 5). (**A**) Schematic representation of the experimental study. Bacterial burden in the lungs of immunized mice was assessed 6 weeks after aerosol infection with *Mycobacterium tuberculosis* Erdman. The bacterial load was quantified by enumerating colony-forming units (CFUs) on Middlebrook 7H10 agar plates. (**B**) Lung CFU counts were determined in each group (*n* = 5 per group) six weeks post-infection by enumerating viable bacteria. (**C**) The extent of lung inflammation and tissue damage was evaluated through histopathological analysis. Data are presented as mean ± SEM. Statistical significance was determined by comparison to the normal control group (* *p* < 0.05, ** *p* < 0.01, *** *p* < 0.001). (**D**) Representative lung histopathology images (H&E staining) depict inflammation and tissue damage across different vaccine groups. Unvaccinated mice exhibited severe immune cell infiltration and granuloma formation. The percentage of lung area affected by granulomatous inflammation was calculated using ImageJ software.

Protective efficacy was assessed in C57BL/6 mice exposed to a low-dose aerosol challenge with *M. tuberculosis* Erdman strain (approximately 50–100 CFU per mouse; Figure S2) three weeks following the final immunization. Bacterial burden, measured by CFU, and lung inflammation were assessed 6 weeks post-challenge. BCG vaccination significantly reduced lung CFU counts compared with unvaccinated controls. Similarly, adjuvanted vaccine candidates were associated with reduced bacterial burden and lung inflammation in *M.tb* Erdman strain-infected mice. Mice immunized with the TLR4 agonist-adjuvanted vaccines showed a more pronounced reduction in CFU counts, with lower bacterial loads compared to those in the control group. Ag85B + QTP709-1 and Ag85B + QTP709-3 groups showed a trend toward reduced CFU counts compared to the BCG-only group; however, no statistically significant differences were observed among the groups (Figure 5B).

To assess vaccine-induced protection against pulmonary inflammation, lung sections from infected mice were processed for histopathological analysis and stained with hematoxylin and eosin to evaluate tissue damage, inflammatory cell infiltration, and structural alterations (Figure 5D). Unvaccinated control mice exhibited severe lung pathology characterized by extensive inflammatory cell infiltration, granuloma formation, and alveolar destruction. In contrast, vaccinated mice displayed reduced inflammatory cell infiltration and preserved pulmonary architecture, suggesting a protective effect against M. tb Erdman-induced lung damage. Among the tested vaccine formulations, mice immunized with the QTP709-1-adjuvanted vaccine exhibited the least lung inflammation, with fewer granulomas and lower inflammatory scores than the control mice. The QTP709-1 group showed notable protection; however, mild inflammation was observed. Analysis of the inflammatory score revealed a correlation between bacterial burden reduction and reduced lung pathology (Figure 5C).

These findings suggest that the tested vaccine formulations confer varying degrees of protection against M. tb Erdman infection. The QTP709-1 adjuvant formulation was associated with effective bacterial control and reduced lung inflammation, suggesting that QTP709-1 may serve as a promising immunostimulatory component for TB vaccine development.

## 4. Discussion

This study examined the immunogenicity and protective efficacy of Ag85B-based subunit vaccine formulations incorporating synthetic TLR4 and STING agonists within a BCG-boosted tuberculosis model. Among the tested adjuvants, the TLR4-based formulations showed improved immunogenicity and protective efficacy compared to the STING-based formulation, and QTP709-1 exhibited a trend toward lower CFU counts compared to the BCG-only group in the M. tb Erdman challenge model.

To elucidate the immunological mechanisms underlying this enhanced efficacy, we first examined innate immune activation, focusing on DC maturation as a key step in initiating adaptive immunity. The QTP709-1 adjuvant was associated with enhanced vaccine-induced immune activity, characterized by increased dendritic cell maturation, augmented adaptive immune responses, and a bias toward Th1- and Th17-associated immunity. QTP709-1 stimulation was associated with elevated surface levels of the co-stimulatory molecules CD80 and CD86 as well as MHC class II (Figure 2A), markers implicated in antigen presentation and T-cell priming. This is consistent with previous findings demonstrating that pattern recognition receptor agonists, such as TLR4 and STING ligands, promote DC activation, cytokine production, and T-cell-mediated immunity [53]. Consistently, QTP709-1–treated DCs induced numerically greater CD4^+^ T-cell proliferation and increased levels of IL-6, IL-12(p40), G-CSF, MCP-1, and KC—cytokines that bridge innate and adaptive immune responses and are associated with Th1 polarization [54,55]. In addition, QTP709-1 significantly increased the production of CCR5-associated chemokines involved in T-cell and monocyte recruitment, including MIP-1α, MIP-1β, and RANTES, suggesting enhanced immune cell trafficking. CCR5 is a chemokine receptor found on multiple immune cell types, including effector T cells, macrophages, and DCs, and contributes to the regulation of immune cell trafficking toward inflammatory sites [56]. Previous studies have shown that, during infectious conditions, CCR5 contributes to the recruitment of antigen-specific T cells to infected tissues, thereby supporting more effective pathogen control [57]. In addition, CCR5 and its ligands, such as RANTES and MIP-1α, are involved in directing the recruitment of T cells and monocytes to sites of inflammation, thereby influencing both innate and adaptive immune responses in the context of vaccination [58]. In this study, the upregulation of CCR5-associated chemokines observed in the QTP709-1 group may have contributed to enhanced immune cell recruitment to target tissues and to improved protective efficacy of this adjuvanted vaccine formulation. To further investigate the mechanistic relevance of this observation, we examined CCR5 activation.

Immunogenicity assessment further demonstrated that all adjuvanted formulations significantly increased Ag85B-specific antibody titers. QTP709-3 induced the strongest IgG(H + L) and IgG1 responses, whereas QTP709-1 elicited the highest levels of IgG2c, indicating a Th1-biased humoral response. In cellular assays, both STING- and TLR4-based adjuvants significantly increased IFN-γ secretion in lung and spleen lymphocytes, with QTP709-1 showing the greatest response. Furthermore, QTP709-1 vaccination produced the highest frequencies of polyfunctional CD4^+^ and CD8^+^ T cells co-expressing IFN-γ, TNF-α, and IL-2, indicative of potent and protective T-cell responses [59]. To determine whether this effect depended on prior BCG priming, we evaluated the immunogenicity of Ag85B using adjuvant formulations administered without BCG vaccination (Figure S3). QTP709-1-adjuvanted vaccination alone induced robust IFN-γ secretion from both lung and splenic lymphocytes, highlighting the potential of QTP709-1 as a standalone adjuvant platform capable of eliciting potent T-cell responses, even without BCG priming.

Collectively, these findings suggest that the activation of CCR5-associated chemokine pathways during early DC–T cell interactions contribute to the effective orchestration of both humoral and cellular immunity in the QTP709-1 group, thereby supporting its potential as a potent vaccine adjuvant formulation. Furthermore, cytokine profiling of lung lymphocytes and splenocytes revealed a distinct Th1/Th17-biased immune environment following vaccination with QTP709-1 adjuvant. Specifically, the QTP709-1 group exhibited the highest levels of TNF-α, IL-2, and IL-17A in both compartments, with splenocytes showing more pronounced systemic responses. In addition to adaptive cytokines such as IL-6, IL-1α, IL-1β, and IL-12(p40), upregulation of GM-CSF and G-CSF in the QTP709-1 group indicates enhanced myeloid cell activation, contributing to effective antigen presentation and early immune defense [60]. By orchestrating a broad cytokine response, QTP709-1 effectively bridges innate activation with protective Th1/Th17-type adaptive immunity [25].

To further elucidate the mechanisms underlying these potent immune responses, we investigated the early innate events triggered by QTP709-1 stimulation. In DC–T cell co-culture settings, QTP709-1 was associated with markedly increased expression of CCR5 and its ligands—MIP-1α, MIP-1β, and RANTES—molecules implicated in immune cell recruitment and early inflammatory signaling [61]. This observation was consistent with the enhanced immunogenicity observed in the QTP709-1 group, including elevated IFN-γ responses and the induction of Th1/Th17-type cytokines. Collectively, these results indicate that CCR5-driven early immune activation is closely associated with the coordination of robust humoral and cellular immune responses elicited by QTP709-1-adjuvanted vaccination.

In both lung lymphocytes and splenocytes, cytokines involved in the initiation of immune responses—such as IL-6, IL-1α, IL-1β, IL-12(p40), GM-CSF, and G-CSF—were elevated following adjuvanted vaccination. Because immune responses in the lungs are critical for host defense against M. tb, the immunogenicity of lung lymphocytes is important for TB vaccine development. In particular, IL-17A deficiency led to impaired granuloma formation and reduced IFN-γ production in the lungs, resulting in significantly weakened protective immunity against BCG immunizations [62]. These findings suggest that the elevated IL-17A levels observed in this study may be functionally associated with enhanced host protection against *M.tb*.

The presence of IL-12(p40), a subunit of IL-12, further supports the induction of Th1 immunity by enhancing IFN-γ secretion and macrophage activation [63]. IL-6 serves as a key mediator that links innate and adaptive immunity by promoting T cell differentiation and acute-phase responses [64]. IL-1α and IL-1β play crucial roles in initiating adaptive immune response and enhancing antigen-presenting cell activation [65]. GM-CSF and G-CSF contribute to myeloid cell differentiation and mobilization, further enhancing the immune response [66]. In the spleen, GM-CSF plays a crucial role in activating and expanding macrophages and DCs, which are essential for antigen presentation and T cell priming [67]. G-CSF promotes the proliferation and migration of neutrophils from the bone marrow to the spleen, where they contribute to early innate immune defense [68]. This cytokine expression pattern indicates that adjuvant vaccination initiates early immune activation, potentially enhancing the recruitment and function of antigen-presenting cells and contributing to the development of antigen-specific adaptive immunity.

Notably, these immunological effects translate into effective in vivo protection. Following aerosol *M. tb* Erdman challenge, all adjuvanted vaccine groups exhibited significant reductions in pulmonary bacterial burden compared with the unvaccinated control group. The QTP709-1 group demonstrated the most pronounced reduction in CFU counts. Histopathological analysis revealed that QTP709-1 vaccination was associated with reduced lung inflammation, characterized by fewer granulomas and better-preserved alveolar structures. These findings suggest an association among immune activation, bacterial control, and lung pathology outcomes. The STING-adjuvanted vaccine elicited substantial immune responses; however, its ability to reduce bacterial burden and lung inflammation was comparatively low, highlighting the need to further optimize STING-based formulations.

## 5. Conclusions

In summary, the synthetic TLR4 agonist QTP709-1 enhances the efficacy of Ag85B-based vaccines by promoting DC maturation, CCR5-mediated immune cell recruitment, and the induction of Th1- and Th17-type immune responses. The study findings suggest that QTP709-1 acts most effectively as an immune potentiator by enhancing Ag85B-driven DC activation more strongly than the other formulations. This activation appears to reinforce CCR5-mediated signaling, which, in turn, facilitates the expansion of polyfunctional CD4^+^ and CD8^+^ T cells and promotes both humoral and cellular immune responses. These factors likely contributed to the superior protective efficacy observed in vivo against *M. tb* infection. Ag85B was used as a model antigen for single-component vaccine evaluation; however, we study validated QTP709-1 as an adjuvant that can overcome the limited immunogenicity of protein subunit vaccines. Given its ability to induce both humoral and cellular immune responses, this adjuvant platform holds promise for broader applications in the development of next-generation vaccines. This platform can be extended to multivalent vaccine constructs incorporating multiple TB antigens to enhance antigenic breadth. Further studies assessing long-term immune durability, memory responses, and performance in diverse host populations are essential to optimize translational readiness. Together, these findings suggest that QTP709-1 is a promising adjuvant platform for next-generation TB vaccines, providing meaningful insights into the rational design of subunit-based vaccination strategies and supporting ongoing efforts in TB vaccine development.

## Data Availability

The original contributions presented in this study are included in the article. Further inquiries can be directed to the corresponding authors.

## Supplementary Materials

The following supporting information can be downloaded at: https://www.mdpi.com/article/10.3390/vaccines14030214/s1, Figure S1: Morphological changes in dendritic cells following stimulation with Ag85B and synthetic adjuvants; Figure S2: Validation of Initial Bacterial Load One Day After *M. tuberculosis* Erdman Challenge; Figure S3: ELISpot Analysis of IFN-γ Responses Following Triple Ag85B-Adjuvanted Vaccination; Table S1: Analytical quality control attributes of QTP709-1, QTP709-3, and QTP701 oil-in-water (O/W) adjuvant emulsions.

## Abbreviations

The following abbreviations are used in this manuscript:

BCG	Bacillus Calmette–Guérin
CFU	colony-forming unit
TB	Tuberculosis
CCR5	C-C motif chemokine receptor 5
