# Supplementary figures and images for: Immunogenicity and Protective Effects of an Ag85B Tuberculosis Subunit Vaccine Formulated with Synthetic TLR4 Agonists in BCG-Boosted Mice
Source: Vaccines (Basel). 2026 Feb 26;14(3):214. doi: 10.3390/vaccines14030214 (PMC13030416; doi:10.3390/vaccines14030214)

Normal

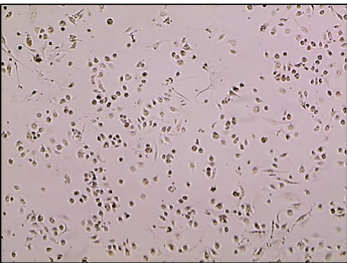

Ag85B

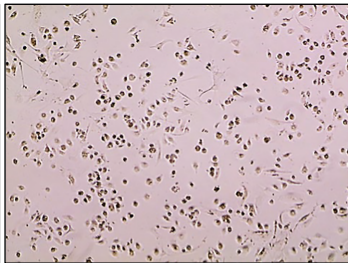

Ag85B + QTP709-1

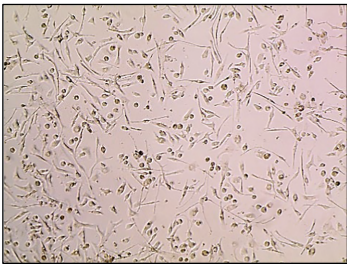

Ag85B + QTP709-3

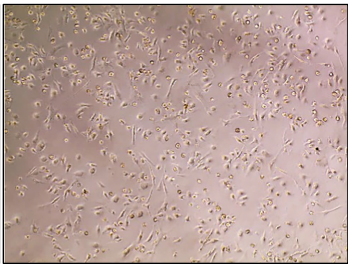

Ag85B + QTP701

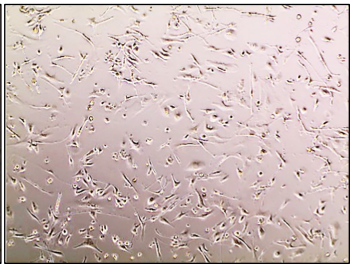

Supplement: Supplementary file 1 [file vaccines-14-00214-s001.zip › vaccines-4067264_Supplementary File(s)/Figure S1.pdf]

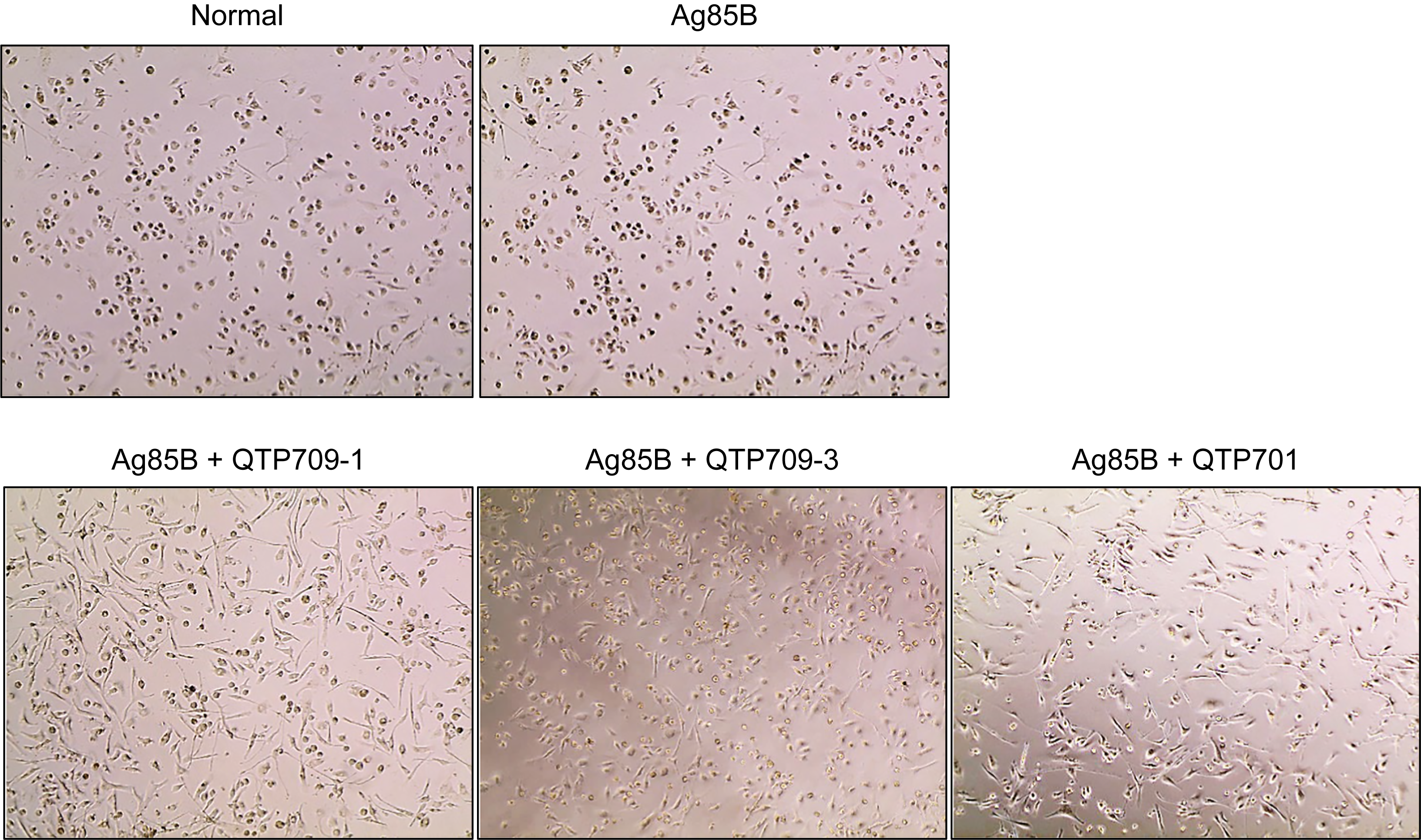

Supplement: Supplementary file 1 [file vaccines-14-00214-s001.zip › vaccines-4067264_Supplementary File(s)/Figure S1.tif]

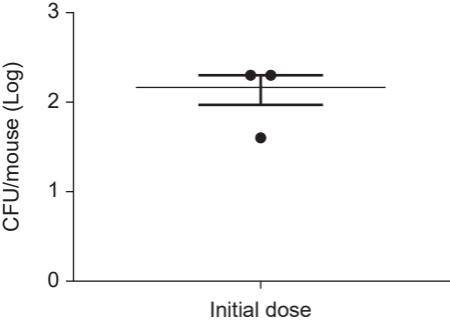

Supplement: Supplementary file 1 [file vaccines-14-00214-s001.zip › vaccines-4067264_Supplementary File(s)/Figure S2.pdf]

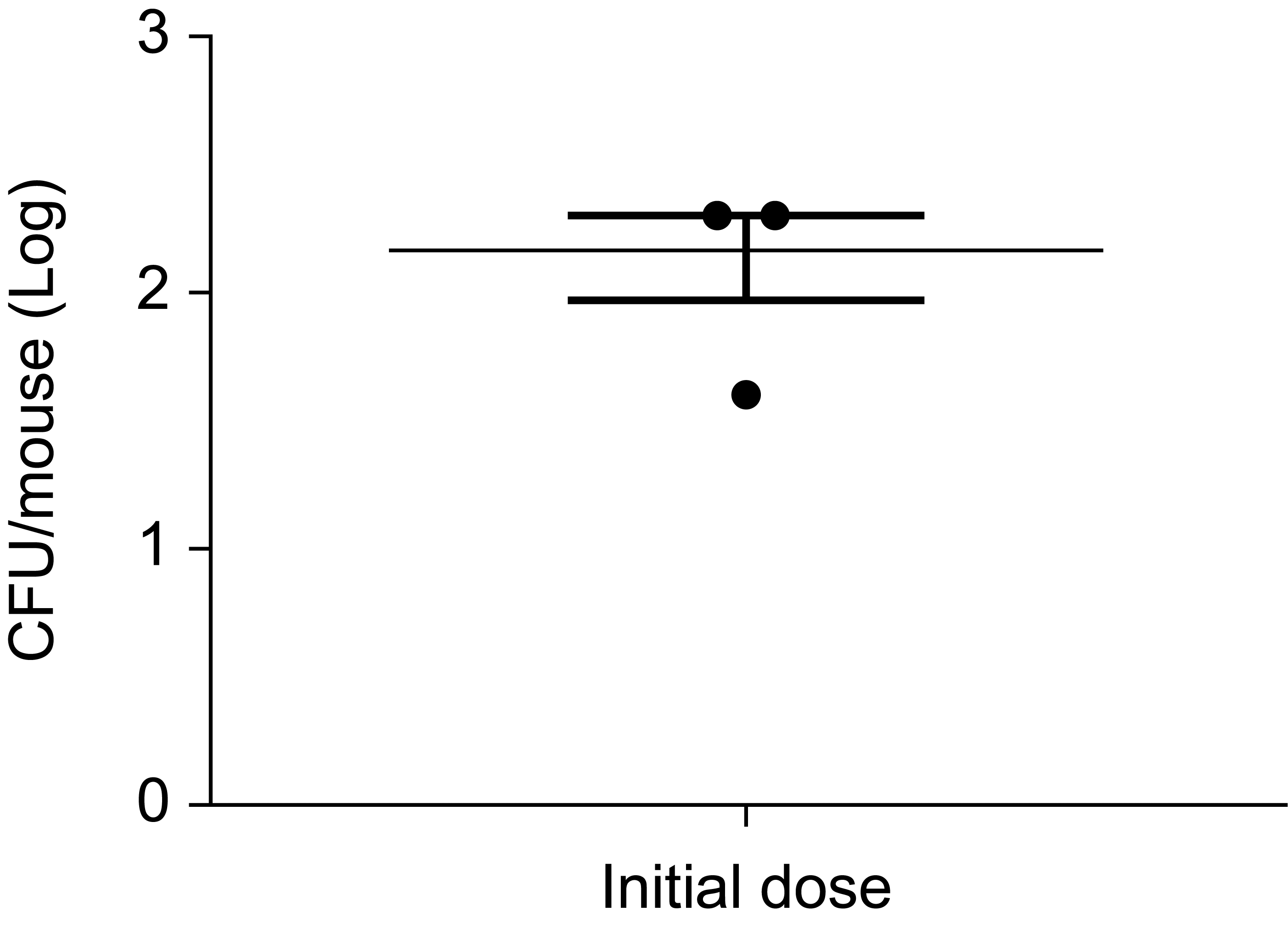

Supplement: Supplementary file 1 [file vaccines-14-00214-s001.zip › vaccines-4067264_Supplementary File(s)/Figure S2.tif]

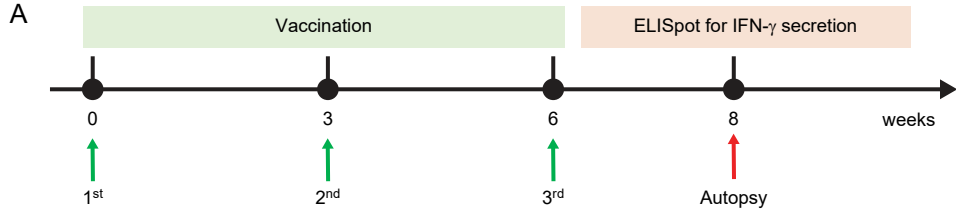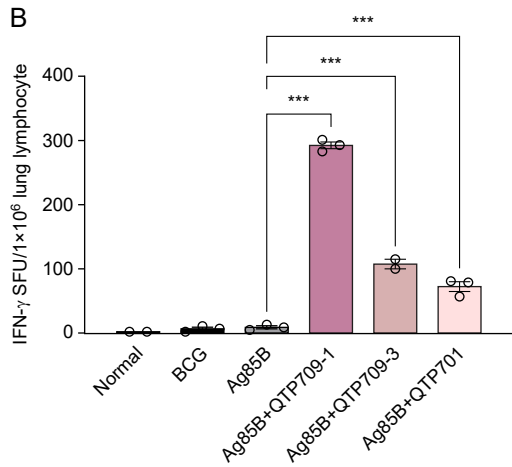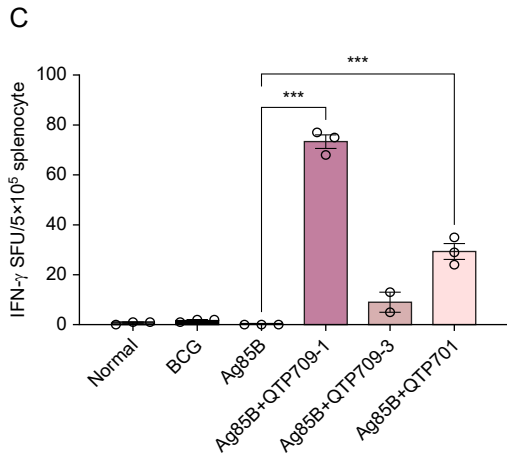

Supplement: Supplementary file 1 [file vaccines-14-00214-s001.zip › vaccines-4067264_Supplementary File(s)/Figure S3.pdf]

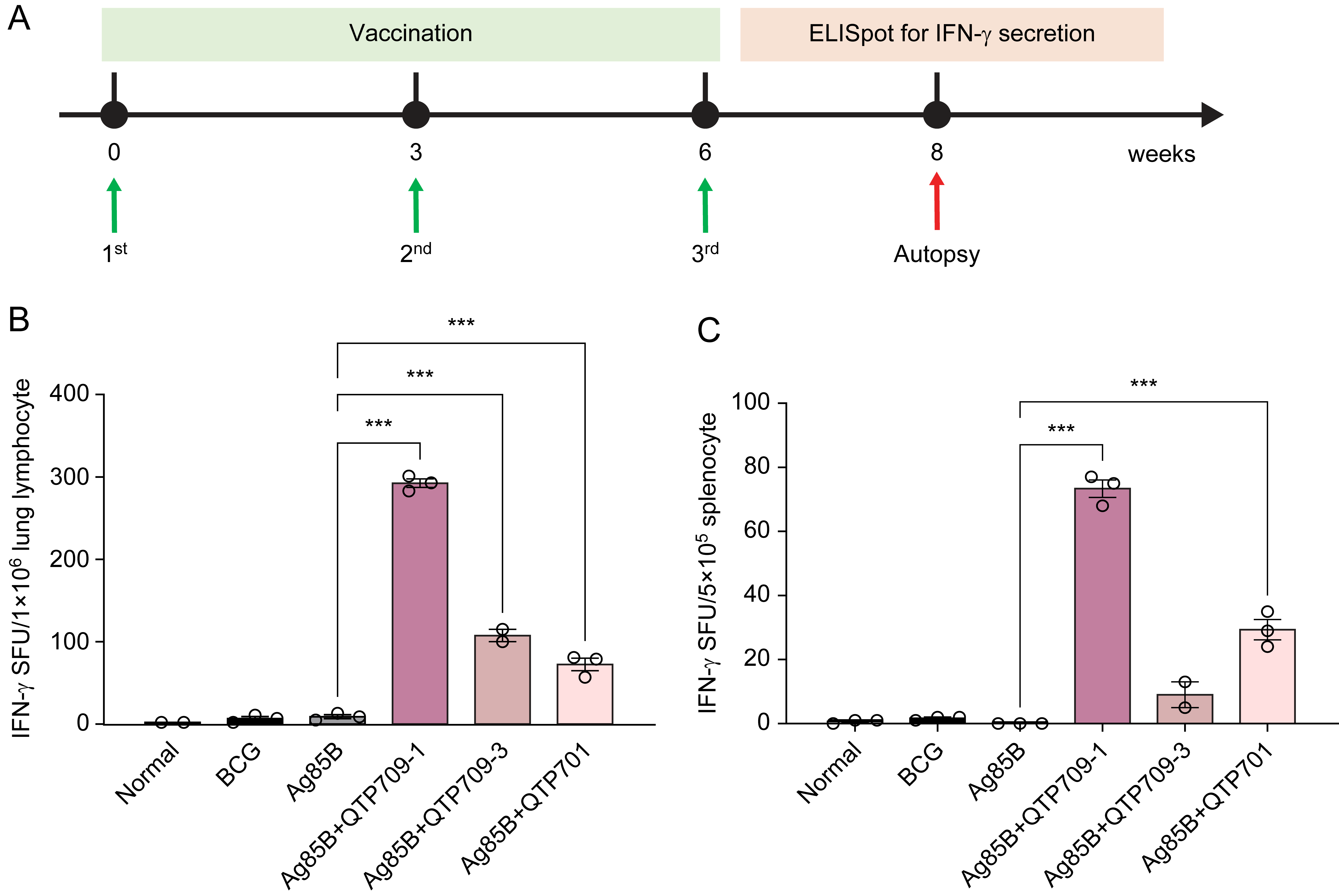

Supplement: Supplementary file 1 [file vaccines-14-00214-s001.zip › vaccines-4067264_Supplementary File(s)/Figure S3.tif]
